# The Moderating Role of Grit in the Relationship Between Perfectionism and Depression Among Chinese College Students

**DOI:** 10.3389/fpsyg.2021.729089

**Published:** 2021-11-05

**Authors:** Jing Zhang, Luming Liu, Wenchao Wang

**Affiliations:** ^1^Research Centre of Applied Technology University, Huanghuai University, Zhumadian, China; ^2^Beijing Key Laboratory of Applied Experimental Psychology, Faculty of Psychology, National Demonstration Center for Experimental Psychology Education (Beijing Normal University), Beijing Normal University, Beijing, China

**Keywords:** perfectionism, grit, depression, college students, moderating role

## Abstract

**Background:** As a personality trait, perfectionism has shown a close association with psychological disorders, such as depression. The protective effect of grit on depression has been confirmed by a series of recent studies. Therefore, it is necessary to investigate the buffering role of grit in the above relationship and the possible underlying mechanism.

**Objective:** Based on the multidimensional theory of perfectionism, we differentiated two dimensions of perfectionism as positive and negative and further examined the relationships between these two dimensions of perfectionism and depression. We also aimed to examine the possible moderating effect of grit on the above two relationships.

**Methods:** Us a questionnaire survey approach, a total of 2,602 college students (1,608 females and 994 males) were assessed using the Frost Multidimensional Perfectionism Scale the Short Grit Scale, and the Center for Epidemiological Studies-Depression Scale. Hierarchical regression analysis was used to investigate the direct effect of two dimensions of perfectionism on depression as well as the moderating role of grit in these associations.

**Results:** After controlling for gender, age, family income, and academic performance, both positive and negative perfectionism had positive predictive effects on depression, and grit negatively moderated these two relationships. Specifically, grit completely counteracted the effect of positive perfectionism on depression yet partly counteracted the effect of negative perfectionism on depression.

**Conclusion:** Grit prevents the depressive symptoms raised by positive and negative perfectionism.

## Introduction

### The Direct Effect of Perfectionism on Depression

Perfectionism, as a stable personality trait, manifests itself in unrealistic standards, overly strict self-evaluation and extreme all-or-nothing thinking, which could result in a series of negative outcomes (Frost et al., 1990). For college students, a cross-cultural meta-analysis revealed that the level of perfectionism has consistently increased since 1989 (Curran and Hill, 2019). Similar to research on perfectionism, research on depression among college students has shown continuous growth, which could be a worrying trend (Gao et al., 2019). College students, who are still in emerging adulthood, have not fully formed coping styles toward difficulties (Heffer and Willoughby, 2017), and facing multiple pressures may lead to a greater possibility of depression affliction (Lan et al., 2019). Therefore, it is of great importance to investigate the possible association between perfectionism and depression in college students.

Regarding the factors that may influence depression, Beck (1986) stated that individual’s cognitive patterns could play a significant role. Following Beck’s cognitive theory of depression, the Integrated Cognitive Model (ICM) took the interaction of dysfunctional attitudes and negative life events as the predictor of depression (Kwon and Oei, 1992). Specifically, dysfunctional attitude referred to a personality trait referring to having an overly rigid cognitive schema of oneself and the world, and perfectionism was one of its major manifestations. From the perspective of theory, perfectionism could be a cause of depressive symptoms, and many related studies have confirmed that (Limburg et al., 2017). For perfectionists, an inappropriate coping style for certain circumstances could result in the arousal of negative emotions such as depression (Tran and Rimes, 2017). Additionally, strict personal standards could lead to higher stress, and the brain association between stress and depression has also been confirmed by cognitive neuroscience (Hankin et al., 2005), which indicates that perfectionism acts as a risk factor for depression *via* stress.

In addition, cluster analysis revealed that perfectionism could be divided into two dimensions: positive and negative (Parker, 1997). Specifically, positive perfectionism was characterized by high personal standards but less concern over mistakes, and negative perfectionism was characterized by extreme concern over mistakes and self-doubt. According to the dual process model explicated by Slade and Owens (1998), for positive perfectionists, the motivation to achieve success allowed them to remain secure emotionally in light of failure, while for the negative perfectionists, the motivation to avoid failure led to the opposite attitude to failure. Furthermore, Dunkley et al. (2003) expressed that positive perfectionism had positive effects by arousing the coping strategies of problem solving, while negative perfectionism conversely had negative effects by arousing the coping strategies of problem evasion. In the Chinese context, the reliability of separating perfectionism into positive and negative parts was also verified. A series of studies have revealed that positive perfectionism was more likely to be associated with positive outcomes and negative perfectionism played an opposite role (Zi and Zhou, 2006; Chen et al., 2013; Wang and Li, 2017).

However, regarding the effect of positive perfectionism on depression, research has shown inconsistent results. While some research has claimed a buffering role of positive perfectionism in depression (Elion et al., 2012), other research showed them to be unrelated (Bergman et al., 2007), indicating that there might be a moderating variable in this relationship. For negative perfectionism, although the present research showed consistent results of the predictive effect on depression (Tran and Rimes, 2017), it is still unclear whether there are possible moderating mechanisms.

### The Moderating Effect of Grit

From the view of positive psychology, the predictive effect of positive coping strategies on depression needs to be discussed (Sin and Lyubomirsky, 2009). According to the diathesis–stress model, the interaction of susceptible traits and buffering mechanisms could act as a predictor of psychological disorders; that is, if individuals have risk traits for psychological problems but develop efficient coping methods for negative events, the possibility of psychological disorders emerging could be greatly reduced (Monroe and Simons, 1991). For the present relationship, grit might be a moderator buffering the negative outcomes caused by perfectionism.

Grit is a stable positive personality trait, defined as perseverance and passion for long-term goals, comprising consistent interests and perseverance. For the relationship of perfectionism and grit, the present literature showed inconsistent viewpoints. Conceptually, grit symbolizes a long-term and passionate commitment, implying a more open mind toward all possible obstacles, while perfectionism implying the obsession with the unrealistic high standard and the ideal outcomes, which was the opposite of grit. However, considering the multidimensions of perfectionism, the positive and negative factors might play different roles in the possible relationship above. For example, in the positive level, the self-oriented perfectionism prediction on conscientiousness and in the negative level, socially prescribe perfectionism showed prediction on neuroticism trait (Stoeber et al., 2009). Meanwhile, grit is also positively correlated with conscientiousness and negatively correlated with neuroticism (Duckworth et al., 2007), and all the evidence above indicating the possible relationship between perfectionism and grit.

Besides, grit can closely influence one’s attitude toward failure and misfortune (Duckworth et al., 2007), implying the possible inner association between grit and adverse mental processes. A meta-analysis confirmed the negative correlation between grit and negative emotional outcomes such as depression (Credé et al., 2017), and a series of previous studies implied the possible buffering role of grit in depression caused by negative personality traits (Musumari et al., 2018). First, from the perspective of present grit theory, when pursuing long-term goals, individuals with higher grit levels tend to maintain persistence and passion while facing obstacles (Duckworth et al., 2007). Thus, grit could help individuals form the belief that failure is unavoidable when striving for success and eventually resist the sense of discrepancy raised by high personal standards (Datu et al., 2018). In addition, cognitive neuroscience has also determined the deeper connection between grit and depression in the brain. A series of studies have confirmed that grit corresponds to the prefrontal cortex (PFC) (Wang et al., 2016). At the same time, dysfunction of the PFC also showed an undeniable connection with self-criticism caused by perfectionism (Longe et al., 2010). Specifically, dysfunction of the PFC could have a negative influence on one’s numerous emotion-related functions, such as self-evaluation and stress coping, which could eventually lead to depression (Dixon et al., 2017), and grit could restrict the process described above. In addition, for individuals with high grit levels, a more optimistic attribution style might occur when facing negative outcomes (Duckworth et al., 2009), which might promote and alleviate the intrinsic effects of positive and negative perfectionism on depression by adjusting cognitive patterns (Flett et al., 1998).

Based on the discussion above, there is evidence for the buffering effect of grit on the relationship between perfectionism and depression. Meanwhile, considering the diverse influences of perfectionism’s two dimensions on depression, differentiation of grit’s moderating effect on the two relationships might be possible. As a result, the two dimensions of perfectionism need to be investigated to further clarify the relationship between perfectionism, grit, and depression.

### Present Study

Based on the ICM, the present study took a dialectical perspective, aiming to examine both the positive and negative influences of perfectionism on depression. Meanwhile, following the view of positive psychology and diathesis–stress model, grit, which is regarded as a positive and preventive personality trait, was also investigated to determine whether it could promote and buffer the positive and negative outcomes produced by two dimensions of perfectionism. Based on relevant theory and former studies, two hypotheses were proposed. Hypothesis 1: In a group of Chinese college students, positive perfectionism acts as a negative predictor, and negative perfectionism acts as a positive predictor of depression. Hypothesis 2: Grit moderates the relationship between the two dimensions of perfectionism and depression. However, it should be noted that a previous study confirmed the influence of gender, age, and family income on depression (Lu et al., 2015). Additionally, several studies have revealed the influence of grit on academic performance (Wang et al., 2016). Because of these findings, the demographic variables above were considered covariates in the following study design.

## Materials and Methods

### Participants

Using the convenience sampling method, a total of 2,602 college students from several universities in China took part in the survey, which was conducted in November 2019. Excluding 8 participants who chose not to report their age, the mean age of the participants was 19.52 (*SD*=1.38), with a range of 17–29years. A total of 1,608 participants (61.8%) were female, while 994 participants (38.2%) were male. The purpose of the survey was explained, and informed consent was obtained from the participating students. The study was approved by the Research Ethics Committee of Beijing Normal University, which confirmed that all research processes performed in this study were in accordance with the ethical standards.

### Measures

#### Perfectionism

The Frost Multidimensional Perfectionism Scale (FMPS; Frost et al., 1990) was used to assess the level of participants’ perfectionism. The Chinese version of FMPS was revised by Zi and Zhou (2006), which showed good reliability and validity in the Chinese participant group. The 25-item instrument consists of the following five subscales: concern over mistakes (Cronbach’s alpha=0.92), parental expectations (Cronbach’s alpha=0.85), personal standards (Cronbach’s alpha=0.87), doubts about actions (Cronbach’s alpha=0.82), and organization (Cronbach’s alpha=0.93). Of these, organization is an indicator of positive perfectionism, while concern over mistakes, parental expectations, personal standards, and doubts about actions compose the negative perfectionism (Parker, 1997). Responses are rated on a 5-point Likert-type scale ranging from strongly disagree (1) to strongly agree (5). Item scores for the two dimensions were added to generate a total score of positive and negative perfectionism, and a higher score indicates a higher level of positive or negative perfectionism.

#### Grit

The Short Grit Scale (Grit-S) was used to assess the participants’ ability to persevere and sustain passion for long-term goals. The Chinese version of Grit-S was revised by Wang et al. (2016), which has good reliability and validity for Chinese participants. For all eight items, responses are rated on a 5-point Likert-type scale ranging from strongly disagree (1) to strongly agree (5). Previous research has indicated that the Grit scale has good reliability and validity (Duckworth et al., 2009). In the current study, Cronbach’s s alpha was 0.95.

#### Depression

The Center for Epidemiological Studies-Depression Scale (CES-D; Radloff, 1977) was used to assess participant levels of depressive symptoms. The Chinese version of CSE-D was revised by Chen et al. (2009), having good reliability and validity in Chinese culture. For all 20 items, responses are rated on a 4-point Likert-type scale ranging from never (0) to always (3). In the current study, Cronbach’s s alpha was 0.96.

#### Data Analysis Strategies

Data were analyzed using IBM SPSS version 26.0 and Interaction version 1.7. The results of Harman’s single-factor test suggested that the variance for both rotated and unrotated first factors was below the threshold of 40%, which indicated that there was no significant common method bias in the study samples. Descriptive statistics were calculated to clarify the preliminary relationship between variables. Based on the descriptive statistics, the moderating effect of grit on the relationship between perfectionism and depression was examined by hierarchical regression analysis, and the significance of moderating effects was further confirmed by a simple slope test.

## Results

### Descriptive Statistics and Correlations

The means and standard deviations of all variables and the intercorrelations among all variables are presented in Table 1. According to the results, first, demographic variables were correlated with psychological variables to different extents. In addition, a significant positive correlation was established between negative perfectionism and depression, while there was no significant correlation between positive perfectionism and depression. In addition, grit was positively correlated with positive perfectionism but negatively correlated with negative perfectionism and depression.

**Table 1:** Means, standard deviations, and correlations among all study variables.

Variables	*M±SD*	1	2	3	4	5	6	7	8
1. Gender^a^	–	1							
2. Age	19.52±1.38	0.00	1						
3. Family Income^b^	–	0.00	−0.10^***^	1					
4. Academic Performance^c^	–	0.11^***^	0.05^**^	0.07^**^	1				
5. Positive Perfectionism	19.40±5.78	0.25^***^	−0.04^*^	0.05^**^	0.15^***^	1			
6. Negative Perfectionism	44.91±16.40	0.11^***^	−0.04	0.00	0.07^***^	0.56^***^	1		
7. Grit	26.55±4.33	0.05^*^	−0.01	0.04	0.16^***^	0.28^***^	−0.11^***^	1	
8. Depression	34.31±8.05	0.00	0.06^**^	−0.07^**^	−0.08^***^	−0.03	0.43^***^	−0.36^***^	1

a *male=1, female=0; ^b^ mean the range of the participants’ Family Income; ^c^ means the range of participants’ Academic Performance (which were represented by average academic score in college)*.

b *below ¥1,000=1, ¥1,000–¥3,000=2, ¥3,000–¥6,000=3, ¥6,000–¥10,000=4, ¥10,000–¥15,000=5, ¥15,000–¥20,000=6, beyond ¥20,000=7*;

c average academic score 0–59=1, 60–69=2, 70–79=3, 80–89=4, 90–100=5. ^*^p<0.05, ^**^p<0.01, and ^***^p<0.001.

### Moderating Analysis

Hierarchical regression analysis was used to examine the moderating effect of grit on the relationship between perfectionism and depression, and the results are displayed in Table 2. The results indicated that the age, family economic situation, and academic performance of college students had significant predictive effects on depression (*β*=0.06, *p*<0.01; *β*=−0.06, *p*<0.01; *β*=−0.08, *p*<0.001), while gender had no influence on depressive symptoms (*β*=0.01, *p*>0.05). Meanwhile, positive and negative perfectionism had significant predictive effects on depression (*β*=0.09, *p*<0.001; *β*=0.41, *p*<0.001), and grit also significantly predicted depression in both relationships (*β*=−0.38, *p*<0.001; *β*=−0.31, *p*<0.001). Furthermore, the interactions of both positive and negative perfectionism with grit acted as significant predictors of depression (*β*=−0.09, *p*<0.001; *β*=−0.09, *p*<0.001).

**Table 2:** The moderating effect on the relationship between perfectionism and depression.

Variables	*F*	*B*	*SE*	*β*	*t*
Step 1	9.43^***^				
Gender		0.08	0.33	0.01	0.25
Age		0.36	0.12	0.06	3.13^**^
Family Income		−0.36	0.13	−0.06	−2.81^**^
Academic Performance		−0.79	0.19	−0.08	−4.20^***^
Step 2	74.14^***^				
Positive Perfectionism		0.12	0.03	0.09	4.51^***^
Grit		−0.71	0.04	−0.38	−20.02^***^
Step 2	188.23^***^				
Negative Perfectionism		0.20	0.01	0.41	24.69^***^
Grit		−0.58	0.03	−0.31	−18.35^***^
Step 3	67.34^***^				
Positive Perfectionism × Grit		−0.03	0.01	−0.09	−4.78^***^
Step 3	166.74^***^				
Negative Perfectionism × Grit		−0.01	0.00	−0.09	−5.12^***^

** *p<0.01 and*

*** *p<0.001*.

**Figure 1:**
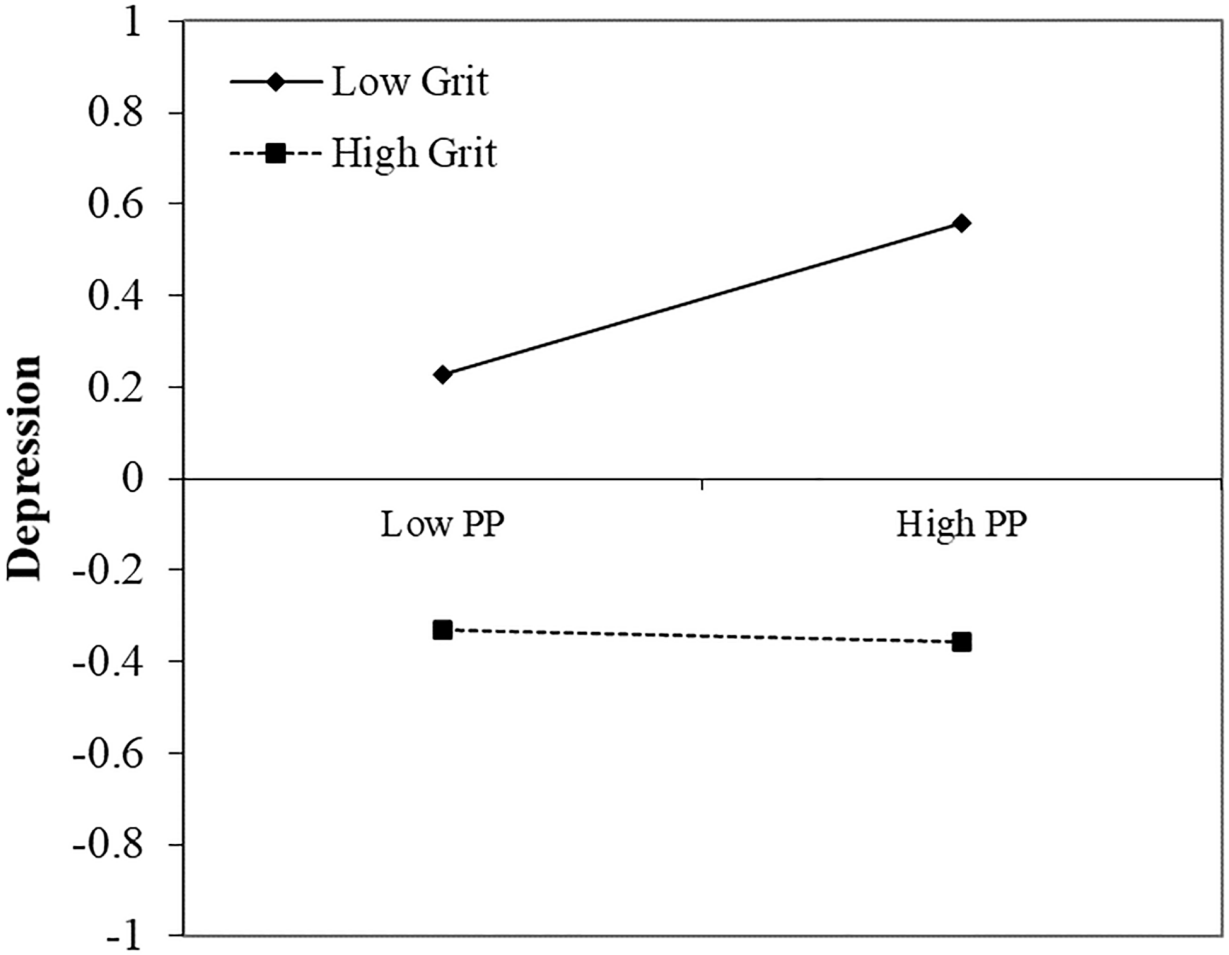
The moderating role of grit on the relationship between positive perfectionism and depression. All the study variables were standardized. PP=Positive perfectionism. High/low level of PP=M±1SD. High/low level of grit=M±1SD.

**Figure 2:**
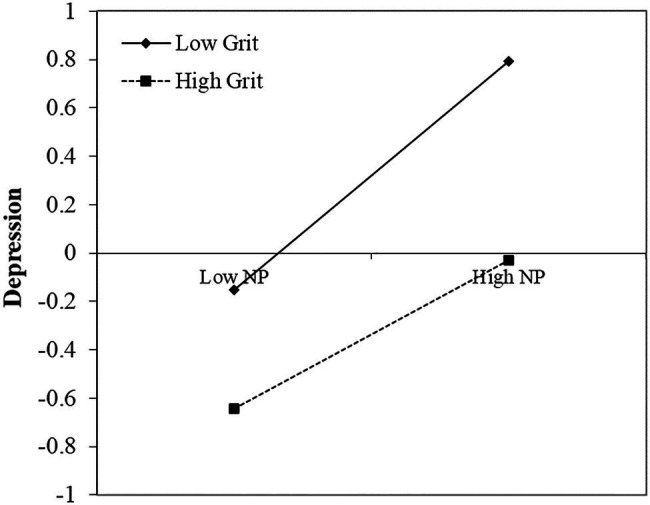
The moderating role of grit on the relationship between negative perfectionism and depression. All the study variables were standardized. NP=Negative perfectionism. High/low level of NP=M±1SD. High/low level of grit=M±1SD.

A simple slope test was used to further examine the significance of the moderating effects, and all the study variables were standardized (see Figures 1, 2). The results indicated that for individuals with a high grit level (1 *SD* above the mean), the predictive effect of positive perfectionism on depression vanished (simple slope=−0.01, *t*=−0.47, *p*>0.05), yet the predictive effect of negative perfectionism on depression remained (simple slope=0.31, *t*=12.51, *p*<0.001). However, for individuals with a low grit level (1 *SD* below the mean), the predictive effect of positive perfectionism on depression was still present (simple slope=0.17, *t*=6.27, *p*<0.001), and a stronger predictive effect of negative perfectionism on depression occurred compared to the individuals with a high grit level (simple slope=0.47, *t*=19.31, *p*<0.001).

## Discussion

Using the mean of hierarchical regression analysis, we established a moderating model to clarify the buffering effect of grit on the relationship between perfectionism and depression. Descriptive statistics indicated different correlation levels between demographic variables and psychological variables, which emphasized the necessity of controlling for covariates. Of note, inconsistent with the hypotheses, positive perfectionism was not significantly correlated with depression. For this unexpected result, a possible explanation could be the limitation of Pearson correlations; that is, they focus only on the two present variables and overlook other influential confounding factors (Fan, 1997). Therefore, further investigation controlling for covariates needs to be conducted to reveal the relationship between the study variables.

### The Direct Effects of Perfectionism on Depression

The results of hierarchical regression analysis indicated that both positive and negative perfectionism positively predicted depression, which was not consistent with previous results. For negative perfectionism, its positive effect on depression was consistent with previous studies (Slade and Owens, 1998); that is, a higher level of negative perfectionism might result in a greater risk of depression symptoms. Dunkley et al. (2003) confirmed that negative perfectionism could mislead college students to ignore personal capacity and develop self-critical attitudes, which ultimately led to unpleasant emotions.

However, unlike the results shown in the related systematic review (Wright et al., 2021), the present study found a positive predictive effect of positive perfectionism on depression. The difference could be attributed to the following. First, for positive perfectionists, the tendency toward organization has a strong relationship with compulsive beliefs (Hollander, 1965) and was thought to be a significant predictor of obsessive–compulsive disorder (Martinelli et al., 2014), and obsessive–compulsive disorder was closely related to depressive symptoms (Goodwin, 2015). As a result, positive perfectionism may raise the level of depression *via* compulsive behavior. In addition, according to an evaluative review of the dual process model, when interpreting the outcome of positive perfectionism, researchers should not overlook the present experience and environment of individuals (Flett and Hewitt, 2006). A related study also revealed that when facing certain situations, such as achievement failure, individuals who were regarded as having traits of positive perfectionism also showed a high risk of depressive symptoms (Hewitt and Flett, 2002).

For college students, the appropriate organization can improve planning skills when facing multiple burdens from academic work and social activities, but the excessive organization may result in unrealistic compulsive beliefs, such as not accepting any change in their plans or magnifying the consequence of any failure. Based on that, the inconsistency of the predictive effect of positive perfectionism on depression with previous studies might be attributed to the different situations that individuals were experiencing.

### The Moderating Effects of Grit on the Relationship Between Perfectionism and Depression

The results of hierarchical regression analysis also showed that grit acts as a moderator between the two dimensions of perfectionism and depression, indicating that grit could be a buffering mechanism against susceptibility traits, which verifies the diathesis–stress model (Monroe and Simons, 1991).

First, as a positive personality trait, grit itself has a preventive effect on depression. Grittier individuals tend to be mindful and have growth mindsets (Li et al., 2018), allowing college students to reconstruct unpleasant experiences and gain new perspectives on academic and peer interactions. Based on this process, students can gain the ability to develop problem-solving strategies to improve learning efficiency and change their inappropriate interpersonal communication patterns, which contributes to relieving negative emotions. In addition, having both grit and perfectionist tendencies can provide great benefits to individuals. Grittier individuals pursue long-term goals, which could compensate for the lack of life meaning caused by perfectionism (Kleiman et al., 2013; Park and Jeong, 2016). Perfectionists in college tend to experience more pressure when meeting strict personal standards for academic performance, and grit could significantly improve their academic achievement to alleviate the mental burden (Rice et al., 2006; Tang et al., 2019).

Meanwhile, the result of the simple slope test is also worthy of attention. Grit entirely counteracts the promoting effect of positive perfectionism on depression, but not the promoting effect of negative perfectionism. The difference can be attributed to the source of perfectionism’s two dimensions. Compared to positive perfectionism, the origin of negative perfectionism contains external factors such as parental expectations. To attain acceptance and love, perfectionists must internalize the high standard set by their parents (Frost et al., 1990), which implies that a positive coping style could not entirely prevent the occurrence of negative emotions at the individual level. Besides, compared to previous studies, the different roles of positive perfectionism are also worth noticing, and the reasons may be attributed to cultural factors. Chinese culture tends to regard the organization as a noble quality and emphasis its necessity, resulting in the education on pursuing organization. However, college students have to face multiple pressure from different sources, which increases the difficulties in achieving high organization in their campus life. The students may have the awareness to be organized but failed due to the lack of grit, and the discrepancy between ideal and reality increased their depression. The relative study also revealed the complicated relationship among perfectionism, grit, and negative academical emotions in Chinese college students (Liu et al., 2021). The present result can also be a possible explanation for positive perfectionism’s different effects in former studies. For grittier individuals, considering the buffering effect of grit, pursuing organization will not increase depression levels.

### General Discussion

Based on the dual process model of perfectionism and the diathesis–stress model, we partitioned perfectionism into positive and negative subtypes and further investigated the buffering effect of grit on the relationship between perfectionism and depression. Verifying and replenishing the previous theories and studies, the present results confirmed the preventive role of positive traits on the adverse outcome caused by negative traits, introducing a new idea to reduce the prevalence of depression among undergraduates.

However, several limitations should also be noted when interpreting the current results. First, since a self-report inventory was used, subjectivity is inevitable, which necessitates that future studies use other data collection methods, such as laboratory experiments. Next, the cross-sectional data lack precision when drawing causal relationships, which could be improved by using a longitudinal design, that is, adding additional time points in future studies. Furthermore, the population scope was limited to college students, which may raise the uncertainty of the external validity, requiring further research in various participant groups. In addition, recent studies have revealed the rationality of separately researching the two dimensions of grit (i.e., consistency of interests and perseverance of effort) (Credé, 2018), so future studies can examine the effects of grit’s two dimensions to further clarify the underlying mechanism. Finally, the internal consistency reliability of variables in the current study was too high, which could result from the potential overlap between items (Tavakol and Dennick, 2011), or the relatively consistent answering patterns of participants when facing specific items. Future studies could take more consideration into the selection of inventories and the correspondence between the inventories and participants.

Despite the limitations above, the merits of the present study are clear for the psychological care of college students. Since grit is a malleable trait (Duckworth, 2016), college students can develop grit to prevent the risk of depressive symptoms. For college students, when facing the choice that will have a long-term impact on the future, they should first consider their interests to identify their passion. In addition, a proper and specific long-term goal should also be set to ensure that they persevere toward it. For those working in psychological counseling, guidance on discovering interests and setting goals for college students should also be noted. Once a high level of grit is cultivated, the depressive symptoms raised by perfectionism can be prevented or alleviated.

## Conclusion

Through the examination of the direct effects of positive and negative perfectionism on depression as well as the moderating effects of grit on the relationship above, we drew the following conclusions. Firstly, in specific situations, both positive and negative perfectionism can act as potential risk factors for depression. Furthermore, grit can significantly prevent the depressive symptoms raised by positive and negative perfectionism, which emphasized the role of grit in reducing the occurrence of depression among college students.

## Data Availability Statement

The raw data supporting the conclusions of this article will be made available by the authors, without undue reservation.

## Ethics Statement

The studies involving human participants were reviewed and approved by The Research Ethics Committee of Beijing Normal University. The patients/participants provided their written informed consent to participate in this study.

## Author Contributions

JZ developed the study design, drafted the manuscript, and revised the manuscript critically for important intellectual content. ML developed the study design, performed the statistical analysis, and drafted the manuscript. WW participated in and supervised the data collection, assisted in data collection and analysis, and made important modifications to the manuscript. All authors contributed to the article and approved the submitted version.

## Funding

The current study was supported by “The Fundamental Research Funds for the Central Universities” (Beijing Normal University), China (Project No. 2020NTSS02) and Research Center for Integrated Development of Industry and Education of Application-Oriented Institutes in Huanghuai College, Key Research Base of Humanities and Social Sciences in Henan Province.

## Acknowledgments

We much appreciate the participants and research assistants involved in the present research. Moreover, we are grateful to the tutor for his detailed guidance and constructive suggestions.

## Conflict of Interest

The authors declare that the research was conducted in the absence of any commercial or financial relationships that could be construed as a potential conflict of interest.

## Publisher’s Note

All claims expressed in this article are solely those of the authors and do not necessarily represent those of their affiliated organizations, or those of the publisher, the editors and the reviewers. Any product that may be evaluated in this article, or claim that may be made by its manufacturer, is not guaranteed or endorsed by the publisher.

## References

[ref1] Beck A. T. (1986). Cognitive models of depression. J. Cogn. Psychother. 1, 5–38.

[ref2] Bergman A. J. Nyland J. E. Burns L. R. (2007). Correlates with perfectionism and the utility of a dual process model. Personality Individual Differ. 43, 389–399. doi: 10.1016/j.paid.2006.12.007

[ref3] Chen C. Yan T. Lin C. (2013). Perfectionism, self-esteem, and academic procrastination among Chinese university students. Psychol. Dev. Educ. 29, 443–445, 448. doi: 10.16187/j.cnki.issn1001-4918.2013.04.002

[ref4] Chen Z. Yang X. Li X. (2009). Psychometric features of CSE-D in Chinese adolescents. Chin. J. Clin. Psychol., 17, 443–445. 10.16128/j.cnki.1005-3611.2009.04.027.

[ref5] Credé M. (2018). What shall we do about grit? A critical review of what we know and what we don’t know. Educ. Res. 47, 606–611. doi: 10.3102/0013189X18801322

[ref6] Credé M. Tynan M. C. Harms P. D. (2017). Much ado about grit: A meta-analytic synthesis of the grit literature. J. Pers. Soc. Psychol. 113, 492–511. doi: 10.1037/pspp0000102, PMID: 27845531

[ref7] Curran T. Hill A. P. (2019). Perfectionism is increasing over time: A meta-analysis of birth cohort differences from 1989 to 2016. Psychol. Bull. 145, 410–429. doi: 10.1037/bul0000138 29283599

[ref9] Datu J. A. D. Yuen M. Chen G. (2018). Exploring determination for long-term goals in a collectivist context: A qualitative study. Curr. Psychol. 37, 263–271. doi: 10.1007/s12144-016-9509-0

[ref10] Dixon M. L. Thiruchselvam R. Todd R. Christoff K. (2017). Emotion and the prefrontal cortex: An integrative review. Psychol. Bull. 143, 1033–1081. doi: 10.1037/bul0000096, PMID: 28616997

[ref11] Duckworth A. L. (2016). Grit: The Power of Passion and Perseverance. New York, NY: Scribner.

[ref12] Duckworth A. L. Peterson C. Matthews M. D. Kelly D. R. (2007). Grit: perseverance and passion for long-term goals. J. Pers. Soc. Psychol. 92, 1087–1101. doi: 10.1037/0022-3514.92.6.1087, PMID: 17547490

[ref13] Duckworth A. L. Quinn P. D. Seligman M. E. P. (2009). Positive predictors of teacher effectiveness. J. Posit. Psychol. 4, 540–547. doi: 10.1080/17439760903157232

[ref14] Dunkley D. M. Zuroff D. C. Blankstein K. R. (2003). Self-critical perfectionism and daily affect: dispositional and situational influences on stress and coping. J. Pers. Soc. Psychol. 84, 234–252. doi: 10.1037//0022-3514.84.1.234, PMID: 12518982

[ref15] Elion A. A. Wang K. T. Slaney R. B. French B. H. (2012). Perfectionism in African American students: relationship to racial identity, GPA, self-esteem, and depression. Cult. Divers. Ethn. Minor. Psychol. 18, 118–127. doi: 10.1037/a0026491, PMID: 22309503

[ref001] Fan X. (1997). Canonical correlation analysis and structural equation modeling: What do they have in common?Struct. Equ. Modeling: A Multidiscip. J. 4, 65–79. doi: 10.1080/10705519709540060

[ref16] Flett G. L. Hewitt P. L. (2006). Positive versus negative perfectionism in psychopathology: A comment on Slade and Owen’s dual process model. Behav. Modif. 30, 472–495. doi: 10.1177/0145445506288026, PMID: 16723426

[ref17] Flett G. L. Hewitt P. L. Blankstein K. R. Pickering D. (1998). Perfectionism in relation to attributions for success or failure: research and reviews. Curr. Psychol. 17, 249–262. doi: 10.1007/s12144-998-1010-y

[ref18] Frost R. O. Marten P. Lahart C. Rosenblate R. (1990). The dimensions of perfectionism. Cogn. Ther. Res. 14, 449–468. doi: 10.1007/BF01172967

[ref19] Gao W. Ping S. Liu X. (2019). Gender differences in depression, anxiety, and stress among college students: A longitudinal study from China. J. Affect. Disord. 263, 292–300. doi: 10.1016/j.jad.2019.11.121, PMID: 31818792

[ref20] Goodwin G. M. (2015). The overlap between anxiety, depression, and obsessive-compulsive disorder. Dialogues Clin. Neurosci. 17, 249–260. doi: 10.31887/DCNS.2015.17.3/ggoodwin 26487806 PMC4610610

[ref21] Hankin B. L. Fraley C. Abela J. R. Z. (2005). Daily depression and cognitions about stress: evidence for a trait-like depressogenic cognitive style and the prediction of depressive symptoms in a prospective daily diary study. J. Pers. Soc. Psychol. 88, 673–685. doi: 10.1037/0022-3514.88.4.673, PMID: 15796667

[ref22] Heffer T. Willoughby T. (2017). A count of coping strategies: A longitudinal study investigating an alternative method to understanding coping and adjustment. PLoS One 12:e0186057. doi: 10.1371/journal.pone.0186057, PMID: 28982138 PMC5642021

[ref23] Hewitt P. L. Flett G. L. (2002). Perfectionism and stress in psychopathology. In Flett G. L. Hewitt P. L. (Eds.). Perfectionism: Theory, Research, and Treatment (pp. 255–284). Washington, DC: American Psychological Association.

[ref24] Hollander M. H. (1965). Perfectionism. Compr. Psychiatry 6, 94–103. doi: 10.1016/S0010-440X(65)80016-5 14298909

[ref25] Kleiman E. M. Adams L. M. Kashdan T. B. Riskind J. H. (2013). Gratitude and grit indirectly reduce risk of suicidal ideations by enhancing meaning in life: evidence for a mediated moderation model. J. Res. Pers. 47, 539–546. doi: 10.1016/j.jrp.2013.04.007

[ref26] Kwon S. Oei T. P. S. (1992). Differential causal roles of dysfunctional attitudes and automatic thoughts in depression. Cognit. Ther. Res. 16, 309–328. doi: 10.1007/BF01183284

[ref27] Lan X. Wang W. Radin R. (2019). Depressive symptoms in emerging adults with early left–behind experiences in rural China. J. Loss Trauma 24, 339–355. doi: 10.1080/15325024.2019.1586188

[ref28] Li J. Lin L. Zhao Y. Chen J. Wang S. (2018). Grittier chinese adolescents are happier: the mediating role of mindfulness. Personal. Individ. Differ. 131, 232–237. doi: 10.1016/j.paid.2018.05.007

[ref29] Limburg K. Watson H. J. Hagger M. S. Egan S. J. (2017). The relationship between perfectionism and psychopathology: A meta-analysis. J. Clin. Psychol. 73, 1301–1326. doi: 10.1002/jclp.22435, PMID: 28026869

[ref30] Liu C. He J. Ding C. Fan X. Hwang G. Zhang Y. (2021). Self-oriented learning perfectionism and English learning burnout among EFL learners using mobile applications: The mediating roles of English learning anxiety and grit. Learn. Individual Differ. 88:102011. doi: 10.1016/j.lindif.2021.102011

[ref31] Longe O. Maratos F. A. Gilbert P. Evans G. Volker F. Rockliff H.. (2010). Having a word with yourself: neural correlates of self-criticism and self-reassurance. NeuroImage 49, 1849–1856. doi: 10.1016/j.neuroimage.2009.09.019, PMID: 19770047

[ref32] Lu W. Bian Q. Song Y. Ren J. Xu X. Zhao M. (2015). Prevalence and related risk factors of anxiety and depression among Chinese college freshmen. J. Huazhong Univ. Sci. Technol. Med. Sci. 35, 815–822. doi: 10.1007/s11596-015-1512-4 26670430

[ref33] Martinelli M. Chasson G. S. Wetterneck C. T. Hart J. M. Bjorgvinsson T. (2014). Perfectionism dimensions as predictors of symptom dimensions of obsessive-compulsive disorder. Bull. Menn. Clin. 78, 140–159. doi: 10.1521/bumc.2014.78.2.140, PMID: 24870847

[ref34] Monroe S. M. Simons A. D. (1991). Diathesis-stress theories in the context of life stress research implications for the depressive disorders. Psychol. Bull. 110, 406–425. doi: 10.1037//0033-2909.110.3.406, PMID: 1758917

[ref35] Musumari P. M. Tangmunkongvorakul A. Srithanaviboonchai K. Techasrivichien T. Suguimoto S. P. Ono-Kihara M.. (2018). Grit is associated with lower level of depression and anxiety among university students in Chiang Mai, Thailand: A cross-sectional study. PLoS One 13:e0209121. doi: 10.1371/journal.pone.0209121, PMID: 30550597 PMC6294431

[ref36] Park H. J. Jeong D. Y. (2016). Moderation effects of perfectionism and meaning in life on depression. Personal. Individ. Differ. 98, 25–29. doi: 10.1016/j.paid.2016.03.073

[ref37] Parker W. D. (1997). An empirical typology of perfectionism in academically talented children. Am. Educ. Res. J. 34, 545–562. doi: 10.2307/1163249

[ref38] Radloff L. (1977). The CES-D scale: A self-report depression scale for re-search in the general population. Appl. Psychol. Meas. 1, 385–401. doi: 10.1177/014662167700100306

[ref39] Rice K. G. Leever B. A. Christopher J. Porter J. D. (2006). Perfectionism, stress, and social (dis)connection: A short-term study of hopelessness, depression, and academic adjustment among honors students. J. Couns. Psychol. 53, 524–534. doi: 10.1037/0022-0167.53.4.524

[ref40] Sin N. L. Lyubomirsky S. (2009). Enhancing well-being and alleviating depressive symptoms with positive psychology interventions: A practice-friendly meta-analysis. J. Clin. Psychol. 65, 467–487. doi: 10.1002/jclp.20593, PMID: 19301241

[ref41] Slade P. D. Owens R. G. (1998). A dual process model of perfectionism based on reinforcement theory. Behav. Modif. 22, 372–390. doi: 10.1177/01454455980223010, PMID: 9722475

[ref42] Stoeber J. Otto K. Dalbert C. (2009). Perfectionism and the big five: conscientiousness predicts longitudinal increases in self-oriented perfectionism. Personal. Individ. Differ. 47, 363–368. doi: 10.1016/j.paid.2009.04.004

[ref43] Tang X. Wang M. Guo J. Salmela-Aro K. (2019). Building grit: The longitudinal pathways between mindset, commitment, grit, and academic outcomes. J. Youth Adolesc. 48, 850–863. doi: 10.1007/s10964-019-00998-0, PMID: 30788767

[ref44] Tavakol M. Dennick R. (2011). Making sense of Cronbach’s alpha. Int. J. Med. Educ. 2, 53–55. doi: 10.5116/ijme.4dfb.8dfd, PMID: 28029643 PMC4205511

[ref45] Tran L. Rimes K. A. (2017). Unhealthy perfectionism, negative beliefs about emotions, emotional suppression, and depression in students: A mediational analysis. Personal. Individ. Differ. 110, 144–147. doi: 10.1016/j.paid.2017.01.042

[ref46] Wang H. Li J. (2017). Positive perfectionism, negative perfectionism, and emotional eating: The mediating role of stress. Eat. Behav. 26, 45–49. doi: 10.1016/j.eatbeh.2016.12.012, PMID: 28131966

[ref47] Wang S. Zhou M. Chen T. Yang X. Chen G. Wang M.. (2016). Grit and the brain: spontaneous activity of the dorsomedial prefrontal cortex mediates the relationship between the trait grit and academic performance. Soc. Cogn. Affect. Neurosci. 12, 452–460. doi: 10.1093/scan/nsw145, PMID: 27672175 PMC5390743

[ref48] Wright A. Fisher P. L. Baker N. O’Rourke L. Cherry M. G. (2021). Perfectionism, depression and anxiety in chronic fatigue syndrome: A systematic review. J. Psychosom. Res. 140:110322. doi: 10.1016/j.jpsychores.2020.110322, PMID: 33278659

[ref49] Zi F. Zhou X. (2006). The Chinese frost multidimensional perfectionism scale: An examination of its reliability and validity. Chin. J. Clin. Psychol. 14, 560–563. doi: 10.16128/j.cnki.1005-3611.2006.06.003

